# Genome sequence of the bialaphos producer Streptomyces sp. DSM 41527 and two putative phosphonate antibiotic producers Streptomyces sp. DSM 41014 and DSM 41981 from the DSMZ strain collection

**DOI:** 10.1099/acmi.0.000770.v3

**Published:** 2024-04-19

**Authors:** Alina Zimmermann, Imen Nouioui, Yvonne Mast

**Affiliations:** 1 Department Bioresources for Bioeconomy and Health Research, Leibniz Institute DSMZ - German Collection of Microorganisms and Cell Cultures, Inhoffenstraße 7B, 38124 Braunschweig, Germany; 2 German Center for Infection Research (DZIF), Partner Site Tübingen, Tübingen, Germany; 3 Braunschweig Integrated Centre of Systems Biology (BRICS), Rebenring 56, 38106 Braunschweig, Germany; 4 Technische Universität Braunschweig, Institut für Mikrobiologie, Rebenring 56, 38106 Braunschweig, Germany

**Keywords:** Genome sequence, *Streptomyces*, natural products, antibiotic, phosphonate

## Abstract

*Streptomyces* sp. DSM 41014, DSM 41527, and DSM 41981 are three strains from the DSMZ strain collection. Here, we present the draft genome sequences of DSM 41014, DSM 41527, and DSM 41981 with a size of 9.09 Mb, 8.45 Mb, and 9.23 Mb, respectively.

## Data availability

Genome sequence-related data availability is listed in Table 1.

## Announcement

Phosphonates are rare and often bioactive natural compounds, many of which are nowadays used commercially as medical therapeutics, herbicides, or pesticides. In the course of screening for new phosphonate antibiotic-producing strains, numerous actinomycete strains from the DSMZ collection were cultivated and their genomes sequenced. Here, we present the annotated genome sequences of three strains, which harbour phosphonate biosynthetic gene clusters (BGCs), including the known phosphinothricin tripeptide (PTT, phosphinothricyl-alanyl-alanine) [1,2] producer strain formerly designated as *Streptomyces hygroscopicus* SF-1293. PTT, also known as bialaphos, is a potent natural product antibiotic mainly utilised for its herbicidal properties [2]. Despite the longstanding reputation of *S. hygroscopicus* SF-1293 (DSM 41527) as a PTT producer and its deposit in several strain collections, the genome sequence was not publicly available. Furthermore, there was no formal description of this strain in literature and no recognition in the List of Prokaryotic names with Standing in Nomenclature (LPSN) [3]. In addition, two *Streptomyces* strains, DSM 41014 and DSM 41981 were genome sequenced and identified as potential phosphonate-producing strains based on the finding of phosphonate-specific biosynthesis genes in their genomes.

For DNA isolation, DSM 41014, DSM 41527, and DSM 41981 were cultivated on a rotary shaker (180 r.p.m.) for 7 days in ISP two broth medium (DSMZ 65) [4] at 28 °C. Then 500 µl of DNA/RNA Shield buffer (Zymo Research, California, USA) were added to 30–50 mg biomass according to the protocol of MicrobesNG (https://microbesng.com/; Birmingham, B15 2SQ, UK) and shipped to the sequencing facility of MicrobesNG following supplier’s instructions. Extraction, purification, quantitative, and qualitative estimation of the DNA, as well as whole genome shotgun sequencing and assembly of sequence reads were performed by the MicrobesNG facility using an Illumina platform with random-PCR library selection and 250 bp paired end protocol. The following methodology is reproduced from the methods supplied by MicrobesNG (accessed on 4 January 2024: https://microbesng.com/documents/39/Genome_Sequencing_Methods_V20231206.pdf). Five to forty microlitres of the cell suspension were lysed with 120 µl of TE buffer, containing lysozyme (MPBio, USA), metapolyzyme (Sigma Aldrich, USA), and RNase A (ITW Reagents, Spain), with subsequent incubation for 25 min at 37 °C. Proteinase K (VWR Chemicals, Ohio, USA) (ﬁnal concentration 0.1 mg ml^−1^) and SDS (Sigma-Aldrich, Missouri, USA) (ﬁnal concentration 0.5 % v/v) were added and the mixture was incubated for 5 min at 65 °C. Genomic DNA was puriﬁed using an equal volume of SPRI beads and resuspended in EB buffer (10 mM Tris-HCl, pH 8.0). DNA was then quantiﬁed with the Quant-iT dsDNA HS (ThermoFisher Scientiﬁc) assay in an Eppendorf AF2200 plate reader (Eppendorf UK Ltd, United Kingdom) and diluted as appropriate. Genomic DNA libraries were prepared using the Nextera XT Library Prep Kit (Illumina, San Diego, USA) according to the manufacturer’s protocol with the following modiﬁcations: input DNA was increased two-fold, and PCR elongation time was increased to 45 s. DNA quantiﬁcation and library preparation were carried out on a Hamilton Microlab STAR automated liquid handling system (Hamilton Bonaduz AG, Switzerland). The library was sequenced using an Illumina NovaSeq 6000 (Illumina, San Diego, USA) with 250 bp paired-end reads setting and 30× depth of coverage. Reads were adapter trimmed using Trimmomatic version 0.30 [5] with a sliding window quality cut-off of Q15. The MicrobesNG bioinformatic pipeline included Kraken [6], a system for taxonomic assignment of short DNA sequences and the BWA-MEM software for mapping the reads. *De novo* assembly was performed on samples using SPAdes version 3.7 [7]. Reads were submitted to NCBI’s Sequence Read Archive (SRA) and genome assemblies were submitted to NCBI as Whole Genome Sequencing projects and annotated with the NCBI Prokaryotic Genome Annotation Pipeline (PGAP) [8]. If not indicated otherwise, for all software default settings were applied. All genome-sequence-related data can be found in (Table 1).

**Table 1.** Sequencing and annotation data of DSM 41014, DSM 41981, and DSM 41527. Information based on PGAP annotation

	DSM 41014	DSM 41981	DSM 41527
Genome length (bp)	9 090 440	9 233 126	8 452 293
Contigs	656	480	278
Genome coverage	90×	101×	58×
Average G+C content	72.0	72.9	71.1
Coding sequences (CDS)	8195	8069	7477
tRNAs	71	73	67
rRNAs	6, 3, 9 (5S, 16S, 23S)	7, 7, 14 (5S, 16S, 23S)	8, 6, 7 (5S, 16S, 23S)
RNA numbers	92	105	91
N50	156 173	254 073	125 653
Number of reads (Illumina)	1 849 402	2 134 210	1 133 683
Accession number	JAVRFF000000000	JAVRES000000000	JAVRFE000000000
BioProject number	PRJNA1016140	PRJNA1016140	PRJNA1016140
SRA accession number	SRX22999589	SRX22999591	SRX22999590

**Fig. 1.**
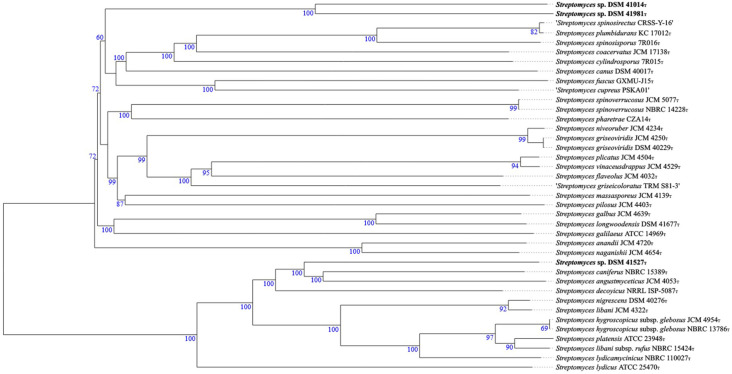
Tree inferred with FastME 2.1.6.1 [19] from GBDP distances calculated from genome sequences. The branch lengths are scaled in terms of GBDP distance formula *d_5_*. The numbers above branches are GBDP pseudo-bootstrap support values >60 % from 100 replications, with an average branch support of 90.7 %. The tree was rooted at the midpoint [20].

The phylogenetic relationship of the strains was analysed with the EzBioCloud database v. 2.1 [9] using the 16S marker gene, which identified DSM 41014 and DSM 41981 as most similar to *Streptomyces cupreus* PSKA01^T^ with 99.52 % similarity [10] and DSM 41527 as most similar to *Streptomyces lydicus* ATCC 25470^T^ with 99.17 % similarity [11]. Phylogenetic relationship based on the full-length genome sequence was carried out with the Type Strain Genome Server (TYGS) v. 1.0 [12,13] (Fig. 1). DSM 41014 and DSM 41981 show closest similarity amongst each other with a digital DNA–DNA hybridization (dDDH) value (formula *d_4_*) of 41.8 % and both share similarity to *Streptomyces cylindrosporus* 7R015^T^ [14] (*d_4_,* 27.3 % and 27.1 %, respectively). DSM 41527 showed phylogenomic relationship to *S. caniferus* NBRC 15389^T^ [15] with a dDDH value (*d_4_*) of 42.4 %. Formula *d_4_* gives values independent of genome length by dividing the sum of all identities found in high-scoring segment pairs (HSPs) with the overall HSP length [16]. A dDDH value below 70 % is indicative for species delineation.

**Fig. 2.**
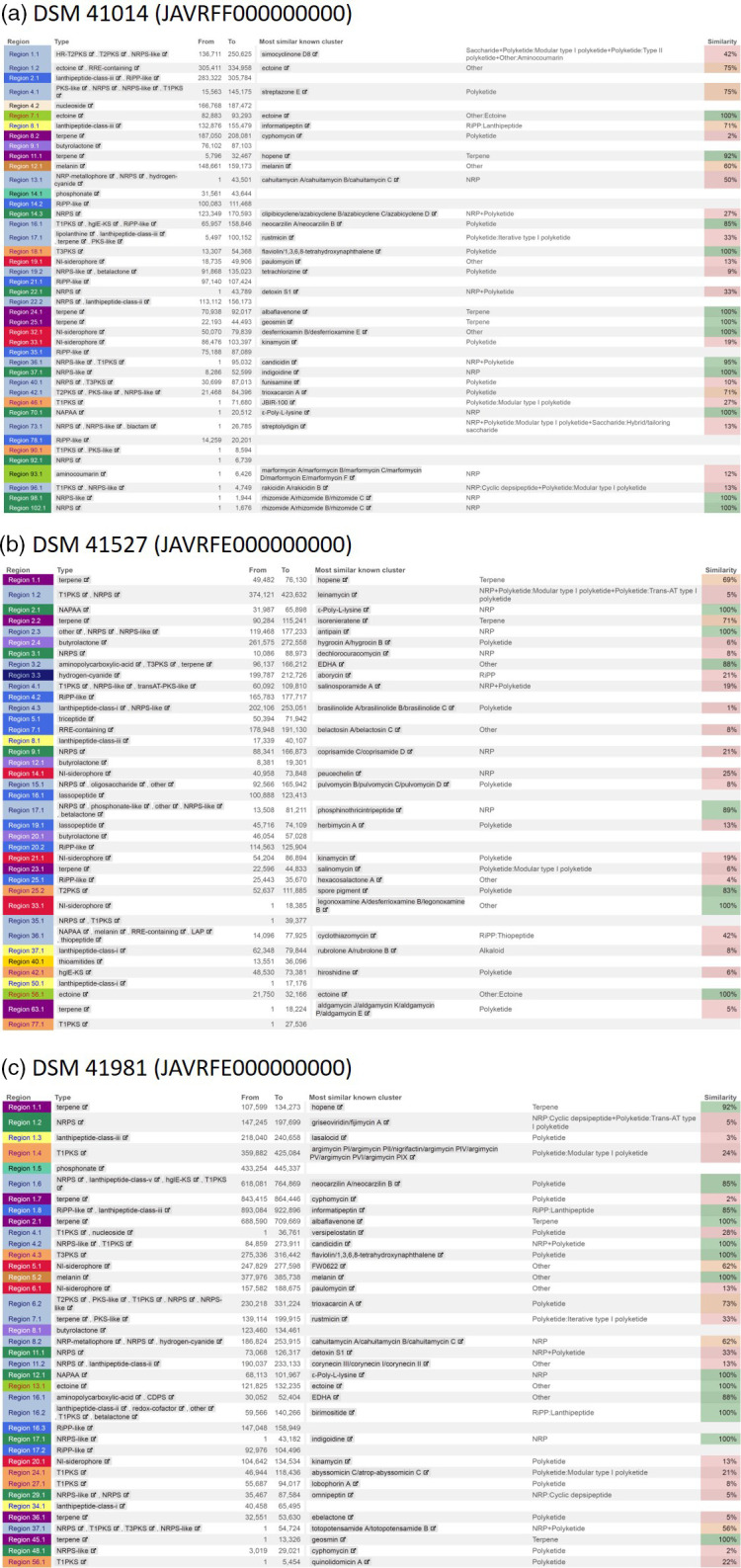
AntiSMASH results from genomes of (a) DSM 41014, (**b**) DSM 41527, and (c) DSM 41981. Phosphonate BGCs are highlighted with a frame.

**Fig. 3.**
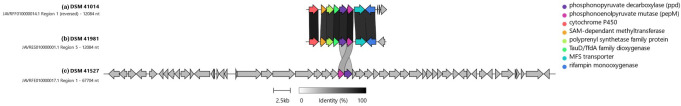
Phosphonate BGCs from (a) DSM 41014, (**b**) DSM 41981, and (c) DSM 41527 (PTT-similar genes). The pepM gene is indicated in pink. Gene cluster comparison was generated with clinker [21].

To unravel the genetic potential for secondary metabolite production, the genome sequences of the three strains were analysed with antiSMASH v. 7.0 [17] for the identification of biosynthetic gene clusters (BGCs). A total of 42, 39, and 38 BGCs were identified for DSM 41014, DSM 41527, and DSM 41981, respectively, with the detection strictness set to relaxed. For all three genomes phosphonate BGCs were identified (Fig. 2a, b, c, respectively), which are characterized by the presence of *pepM*-like genes, known to code for the enzyme phosphoenolpyruvate mutase (PepM), which is the first and essential enzyme of phosphonate biosynthesis [18]. Phosphonate BGCs were identified for DSM 41014 (region 14.1) and DSM 41981 (region 1.5) (Fig. 3a, b, respectively). Both phosphonate BGCs contained the same set of annotated genes, indicating they probably encode for the biosynthesis of similar phosphonate compounds. DSM 41527 contained phosphonate biosynthetic genes as part of a larger hybrid BGC (region 17.1) (Fig. 3c), which included 100 % of all known PTT biosynthetic genes also present in *Streptomyces viridochromogenes* (MIBiG BGC0000406) [2].

Thus, we here present the first draft genome sequence of the bialaphos producer *S. hygroscopicus* SF-1293 (DSM 41527) and disclose the genetic potential of DSM 41014 and DSM 41981 to synthesize phosphonate natural compounds.

## Abbreviations

BGC: biosynthetic gene clusters
dDDH: digital DNA-DNA hybridization
DSMZ: German Collection of Microorganisms and Cell Cultures GmbH
DZIF: German Center for Infection Research
GBDP: genome blast distance phylogeny
HSP: high-scoring segment pairs
ISP: International *Streptomyces* Project
LPSN: List of Prokaryotic names with Standing in Nomenclature
PCR: Polymerase Chain Reaction
PepM: phosphoenolpyruvate mutase
PGAP: Prokaryotic Genome Annotation Pipeline
PTT: phosphinothricin tripeptide
SDS: sodium dodecyl sulfate
SRA: Sequence Read Archive
TYGS: Type Strain Genome Server
